# Redox-Responsive Polymeric Nanogels as Efficient mRNA Delivery Vehicles in *Caenorhabditis elegans*

**DOI:** 10.17912/micropub.biology.001428

**Published:** 2024-12-10

**Authors:** Rupali Dabas, Alan Koh, David Carling, Nazila Kamaly, André E. X. Brown

**Affiliations:** 1 MRC Laboratory of Medical Sciences, Institute of Clinical Sciences, London, England, United Kingdom; 2 Department of Chemistry, Molecular Sciences Research Hub, Imperial College London, W12 0BZ London, United Kingdom

## Abstract

Efficient delivery of sensitive nucleic acid payloads, including mRNA, in *Caenorhabditis elegans* remains challenging, especially with traditional, labor-intensive transgenesis methods. We addressed these challenges using polymeric nanogels (NGs) as an advanced platform for mRNA delivery in *C. elegans* . These polymeric delivery vehicles can be engineered to suit desired applications owing to their chemical versatility, resulting from the ability to conjugate multiple functional groups onto the same backbone. Here, we validate the *in vivo* RNA delivery potential of redox-responsive NGs. The NGs showed up to 72.4 % RNA encapsulation and 6.61 % loading efficiencies and facilitated the controlled release of the mRNA payloads at intracellular concentrations of the reducing agent glutathione, where most of the RNA was released within 24 hours. As a proof of concept, we successfully delivered green fluorescent protein (GFP)-expressing mRNA using NGs in *C. elegans* for the first time. Physicochemical characterization revealed uniform NG size and charge, and fluorescence microscopy confirmed GFP expression in the gut after 24 hours of treatment. Our findings show NGs' potential as an mRNA delivery system in *C. elegans* .

**Figure 1. A fluorescent nanogel (NG) toolkit to mediate mRNA delivery in vivo:**
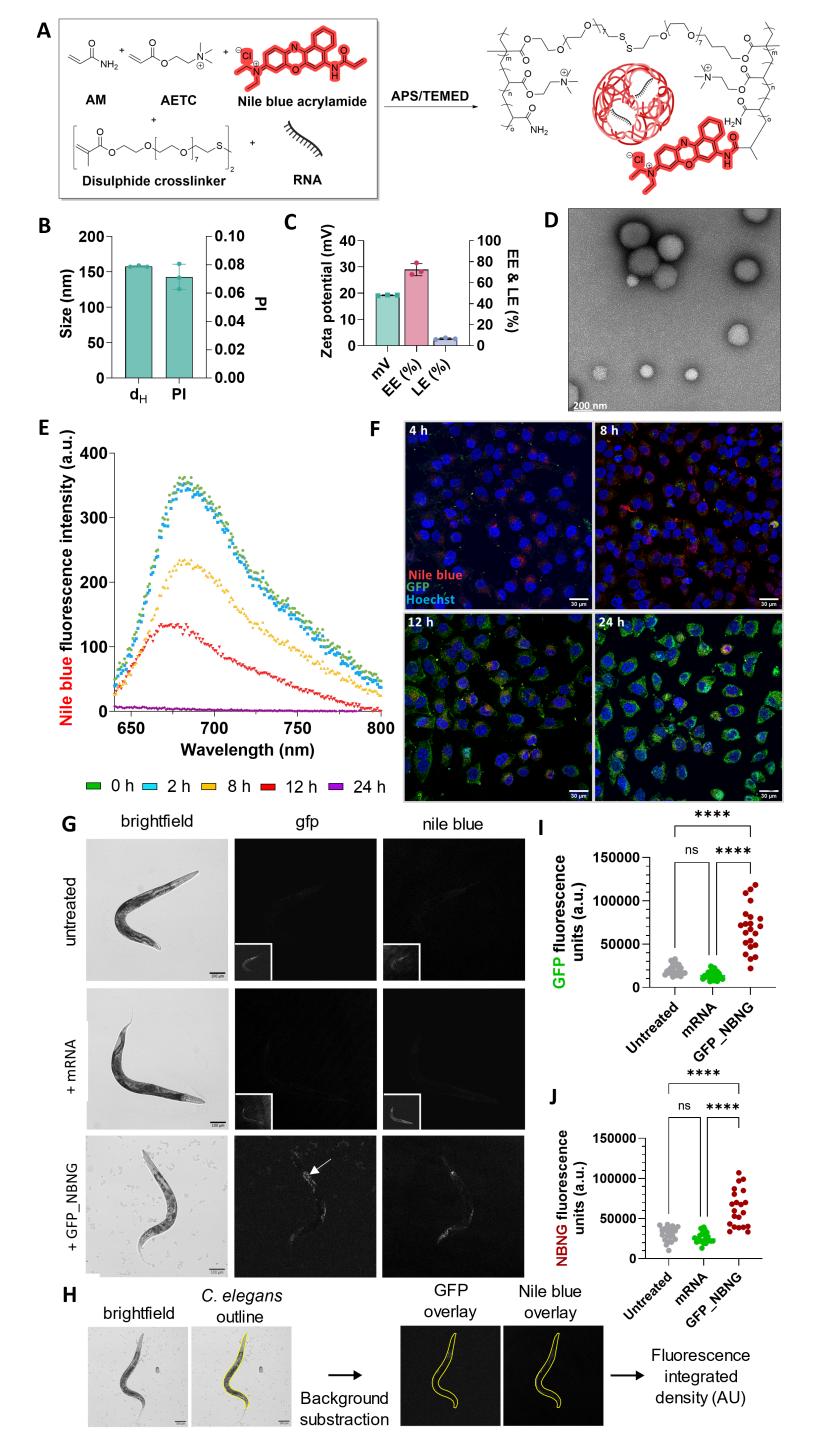
**(A)** Schematic presentation of disulfide-bearing nanogels for mRNA delivery. *Abbreviations: AM: acrylamide, AETC: 2-(acryloyloxy)ethyl trimethylammonium chloride, APS: ammonium persulphate, TEMED: *N,N,N′,N′* -Tetramethylethylenediamine **(B)** Physicochemical characterization of nile blue GFP mRNA-loaded nanogels (GFP_NBNG). Size (hydrodynamic diameter) and polydispersity (PI) as obtained from dynamic light scattering measurements. **(C)** Zeta potential (mV) of synthesised NGs as obtained from electrophoretic scattering measurements (measured in water, pH 7.2) and encapsulation and loading efficiency of GFP mRNA within the NBNGs. **(D)** Negative stain transmission electron micrographs of GFP NBNGs (75,000 x). Scale bar at 50 nm. In all cases, data are presented as mean ± standard deviation for n = 3. **(E)** Emission spectra for degrading NBNGs demonstrates loss of nile blue fluorescence over time. Excitation at 633 nm. **(F)** Confocal microscopy images showing the intracellular uptake and degradation of nile blue labelled (red) GFP mRNA loaded NBNGs in HeLa cells. Cells were stained with 1 µg/mL Hoechst 33342 (blue). All images were taken with 40 x oil immersion objective. Scale bar at 30 μm. **(G)** Confocal brightfield (left panels), GFP fluorescence (middle panels) and nile blue fluorescence (right panels) images of *C. elegans* . Depicted are representative day 2 adult *C. elegans* treated with either NG (400 µg/ml), mRNA encoding GFP (4 µg/ml) or GFP mRNA loaded NBNGs (400 µg/ml) in nematode growth medium (NGM) at 20 °C for 18-24 h. GFP expression in the gut is indicated by the white arrow. The large, depicted images preserve the fluorescence intensity differences between the individual conditions. The small insert images serve to visualize the presence of animals in otherwise dark image fields. **(H)** Fluorescence intensity of individual *C. elegans* were measured by tracing the outline of the animals in brightfield, followed by overlaying the outline onto GFP and nile blue fluorescence micrograph after background subtraction. **(I-J)** Quantification of GFP and nile blue fluorescence intensity of *C. elegans* as shown by the representative images in (G), n = 20 to 25 from one representative biological replicate. Median intensities are indicated with red lines, together with P values of an unpaired, two-sided tests, **** P < 0.0001, ns = not significant.

## Description

*Caenorhabditis elegans* serves as a powerful model for genetic studies, particularly in developmental genetics, cell biology and evolution (Blaxter, 2011; Larsen et al., 2017) . Delivering mRNAs to test protein function in *C. elegans* would be useful, but delivering nucleic acids requires labor-intensive methods, including microinjection or microparticle bombardment for delivery (Ghanta et al., 2021; Schweinsberg and Grant, 2013) . A feeding-based method for mRNA delivery similar to that used for delivering double stranded RNA for RNA interference would make it faster and easier to test proteins for their function *in vivo* . While a variety of transduction strategies for nucleic acids, have been well established for mammalian cells, such protocols are not readily applicable to *C. elegans* (Adams et al., 2019; Kage-Nakadai et al., 2012; Perni et al., 2017) . The thick cuticle of the skin and the acidic pH in the gut of the worms can complicate nucleic acid administration (Perni et al., 2017) . While electroporation has been used for transfecting worms on a population-wide level, the resultant high mortality and low efficiency remain a limitation (Khodakova et al., 2021) . Here, we propose the use of polymeric nanogels (NGs) as a platform for mRNA delivery in *C. elegans* . NGs are colloidal hydrogel particles made up of three-dimensional, cross-linked hydrophilic polymer networks, and represent a relatively new and promising class of delivery systems (Kabanov and Vinogradov, 2009) . They are highly water-swellable, allowing mesh-size-dependent diffusion of encapsulated payloads, which are protected from degradation by the polymer matrix. Due to their chemical flexibility, NGs can be synthesized using combinatorial, free-radical polymerisation of various polyethylene glycol (PEG)-based ionisable, cationic, or stimuli-responsive groups (Cheng et al., 2011; Kabanov and Vinogradov, 2009) . This enables efficient entrapment and controlled spatiotemporal release of mRNA. Additionally, NG surfaces can be modified to extend blood circulation time, improve cellular uptake, and enhance endosomal escape. NGs have already shown success in delivering a range of RNA payloads (Adamo et al., 2016; Chen et al., 2019; De Backer et al., 2015; Duskunovic et al., 2023; Khaled et al., 2016; Zilkowski et al., 2019) . Our strategy involves exploiting intracellular redox balance by engineering NGs that degrade at cytosolic glutathione (GSH) concentrations (2-10 mM), allowing controlled mRNA release within cells (Dabas and Kamaly, 2024) . By incorporating a glutathione-cleavable, disulfide-based crosslinker, we demonstrate NG-mediated delivery and redox-responsive release of functional mRNA.

As a proof of concept, we delivered mRNA coding for GFP by feeding NGs to *C. elegans* ( Figure 1A ). By entrapping the GFP mRNA in NGs, we hypothesise that the RNA will be shielded from degradation in the acidic environment in the digestive tract of the worms. To track the NGs and the RNA independently, we labelled the NGs with a far-red fluorophore (nile blue acrylamide ex/em: 635/674 nm). The physicochemical characterisation of the nile blue-labelled NGs (GFP_NBNG) demonstrated consistent sizes (157.83 ± 1.01 nm; PDI: 0.07 ± 0.01) ( Figure 1B,D ) and surface charge (19.18 ± 0.14 mV) ( Figure 1C ). A 72.67% RNA encapsulation efficiency (EE %) and 7.17% loading efficiency (LE %) indicate that the nanogels effectively capture a substantial amount of RNA, with a moderate payload density that can support stable and controlled release in vivo. ( Figure 1C ). We first confirmed the cellular uptake ability of the NGs in HeLa cells ( Figure 1E -F). By conducting confocal microscopy at specific time intervals, we identified a 24 h window for NG degradation, as evident in the decreasing nile blue fluorescence intensity, which is presumed to dissipate due to removal of degraded NGs from the cells ( Figure 1E ). We also confirmed that this correlated with increasing GFP expression as evident in the increasing green fluorescence at the 24 h timepoint in the confocal microscopy images ( Figure 1F ).

For *in vivo* transduction, we incubated worms in liquid NGM supplemented with 400 µg/mL GFP_NBNG. Under these conditions, we believe the animals ingest the NGs, which then fuse with the walls of the intestinal lumen and are subsequently absorbed into the body, releasing their cargo. From our *in vitro* experiments in HeLa cells, we confirmed NG uptake and subsequent GFP expression within 24 h. Therefore, we conducted confocal microscopy on immobilized live worms after a 24 h incubation period. We observed GFP expression in the gut for worms treated with the GFP_NBNG formulation, whereas the untreated and GFP mRNA controls did not demonstrate any such fluorescence ( Figure 1G -J). We observed a statistically significant increase in both GFP and nile blue signal in the worms treated with the GFP_NBNGs after 24 h. These results confirmed that functional GFP mRNA was delivered into the gut of the worms following encapsulation within the NBNGs. Interestingly, in areas where we observed the GFP signal, we did not observe a nile blue signal, which could reflect the degradation of the NGs. The observed degradation might suggest that the nile blue signal diminishes within the physiological pH range, even if disulfide bond cleavage alone does not account for this change. This result merits further investigation as rapid delivery of mRNA by feeding could enable largescale testing of proteins for *in vivo* function.

## Methods

**RNA-loaded NG synthesis.** NG synthesis was conducted as described previously, with a few minor alterations to prevent RNA degradation (Dabas and Kamaly, 2024) . mRNA encoding green fluorescent protein (GFP) was purchased from Aldevron, UK. Prior to the experiment, all equipment was UV-treated and oven-dried overnight to maintain RNAse-free reaction conditions. In addition, all surfaces were treated with RNAseZap™. RNAse-free water was used and was deoxygenated by nitrogen flushing overnight. Briefly, 6 µg of RNA was added to a 1 mL solution containing acrylamide (2 mg, 0.029 mmol, AM), disulfide crosslinker (4.5 mg, 0.004 mmol), and 2-[(acryloyloxy) ethyl] trimethylammonium chloride (2 µL, 0.01 mmol, AETC) solution and this solution was left to stir for 30 min on ice under a continuous nitrogen atmosphere. For fluorescent NGs, nile-blue acrylamide (0.2 mg, 0.005 mmol) was added to the monomer mix and the reaction was kept protected from light. Sodium dodecyl sulfate (8 mg, 0.028 mmol) was then added to enable micelle formation and left to stir for 30 min. Subsequently, to initiate free-radical polymerization ammonium persulfate (2.28 mg, 0.01 mmol) and *N,N,N′,N* ′-tetramethylethylenediamine (2.5 µL, 0.022 mmol) were dissolved in 1 mL of RNAse free deoxygenated water and were added to the reaction flask. The reaction mixture was observed to achieve a pale blue opalescence within 30 min, which indicated reaction completion. The reaction was stopped by exposure to air once the characteristic opalescence was observed. The reaction mixture was then purified by centrifugation (1200 x g, 10 min) using 15 mL 30kDa MWCO Amicon® Ultra-15 centrifugal filter units (Sigma Aldrich, UK) with five washes against RNAse free water. The NGs were stored at 2-8 °C.

**Dynamic Light Scattering.** Dynamic light scattering (DLS) was used to determine the hydrodynamic diameter and polydispersity of the synthesised nanogels (NGs) in RNAse free water (pH 7.4) and was measured using the Zetasizer Nano-ZS instrument at 22 °C, with backscatter detection at 173°. Three scans were performed each time and each run measured 15 number of sub-scanning cycles (n = 3).

**Zeta Potential.** Zeta potential measurements were also conducted at 22 °C using a Zetasizer Nano-ZS instrument using a folded capillary cell in RNAse free water (pH 7.4).

**Transmission Electron Microscopy (TEM).** TEM was used to image each set of NGs synthesised in this study to confirm the results obtained using DLS. Negative stain EM: 3.5 µL of NG suspension in RNAse free water was pipetted directly onto a glow-discharged lacey carbon film grid with 300 mesh copper (Agar Scientific, UK) and left on the grid for 1 min. Then the remainder of the solution was removed gently using a filter paper. The grid was then stained with 3.5 µL of 2 % (w/w) uranyl acetate for 1 min, after which the stain was removed gently using a filter paper. Cryogenic electron microscope (cryo-EM) was used to confirm the presence of RNA within the synthesised NGs and for resolving the structure of the RNA-loaded NGs at near-atomic resolution. 3.5 µL of RNA-loaded NG suspension in RNAse free water (pH 7.4) was applied to a lacey carbon-coated copper TEM grid, blotted automatically for 1-2 seconds using a Vitrobot™ Mark IV Thermo system, and then plunged unto liquid ethane. The grids were stored in liquid nitrogen under imaging. Samples were imaged on a Talos F200X G2 Transmission Electron Microscope at 45,000 x.

**Encapsulation and loading efficiency measurements.** The encapsulation efficiency (EE %) of the RNA-loaded NGs was measured using the Quanti-It RiboGreen RNA assay kit (Invitrogen). In brief, after the NGs were synthesised, any external, uncomplexed RNA was quantified using this assay kit, according to the manufacturer's instructions. The encapsulation efficiency was further calculated using the following formula:

EE (%) = ((total RNA - measured external RNA)/total RNA) x 100%

To calculate the loading efficiency, the NGs were lyophilised and the following formula was used:

LE (%) = (measured RNA mass/total empty nanogel mass) x 100%

**Cell culture.** Hela cells were obtained from ATCC and were cultured in Dulbecco's Modified Eagle's medium (DMEM, Gibco™) supplemented with 10% foetal bovine serum and 1% penicillin-streptomycin (Sigma-Aldrich, UK). Cells were maintained at 37 °C and 5% CO _2_ .

**Bacterial and worm strains**

The following bacterial strain was used:

*E. coli:* OP50

*E. coli* was routinely maintained on lysogeny broth (LB) agar under standard laboratory conditions.

The following worm strain was used:

N2 : (Bristol isolate wild type)

*C. elegans* were maintained on NGM-plates under standard laboratory conditions (Brenner, 1974) .

**Confocal microscopy.** 5 x 10 ^4^ HeLa cells/well were seeded in µ-Slide 8 Well chambered slides (Ibidi, GmbH) overnight. The next day, the media was replaced, and the cells were incubated with 20 μg/mL of nile blue-labelled disulphide NGs for 24 h. Then the cells were washed thrice with PBS and fixed with 4% paraformaldehyde for 10 min on ice. The cells were then washed thrice and stained with Hoechst 33342 (100 ng/mL) in PBS for 15 min. The cells were then washed thrice with PBS and imaged using the Leica TCS SP8 DLS inverted microscope (Leica, Germany) using LASX software (Leica, Germany). Images were analysed using ImageJ (NIH, USA).

**Fluorescence microscopy.** Synchronized *C. elegans* day 1 adults were collected from NGM-plates and washed twice with liquid NGM. A 1 ml of culture containing worms were transferred to 24-well plates (Corning) and treated with either mRNA (4 ug/ml) or GFP mRNA loaded NBNGs (400 µg/ml), supplemented with OP50 as a food source. The worms were then incubated at 20°C for 18-24 h with shaking at 220 rpm. To immobilize worms for microscopy, worms were incubated with levamisole (1 mM; Sigma-Aldrich) for 1 min, then mounted onto a microscope slide (VWR) with a thin layer of 2 % agarose (Sigma-Aldrich) dissolved in H _2_ O. Immediately after the droplet have dried, a clean glass coverslip (VWR) was placed on top and fluorescence microscopy performed. Images were captured using a Leica TCS SP8 DLS inverted microscope (Leica, Germany) using LASX software (Leica, Germany) and analyzed using Fiji (Schindelin et al., 2012) .

**Fluorescence intensity quantification.** Fluorescence intensity was quantified using Fiji by tracing worm outlines in brightfield images. These outlines were subsequently overlaid onto the corresponding fluorescence images following background subtraction and the total integrated fluorescence intensity was obtained ( Figure 1H ).

**Statistical Analysis.** All statistical analyses were performed using GraphPad Prism. The *p* values were obtained by the Student's *t* -test, and the *p* value of <0.05 was considered statistically significant. The data were presented as mean ± standard deviation.
